# Rare Primary Pulmonary Marginal Zone Lymphoma Presenting with Incidental Pulmonary Nodules

**DOI:** 10.1155/2019/7031868

**Published:** 2019-03-20

**Authors:** Saad Ullah, Mirza Ali, Mingchen Song

**Affiliations:** ^1^Division of Pulmonary and Critical Care Medicine, Southern Illinois University, Springfield, IL, USA; ^2^Department of Internal Medicine, Southern Illinois University, Springfield, IL, USA

## Abstract

Pulmonary malignancies carry a significant morbidity and mortality and are one of the leading causes of cancer-related deaths worldwide. Primary pulmonary lymphoma is a rare malignancy which should be considered in the differential of solitary pulmonary nodule or lung mass especially in a low-risk patient presenting with constitutional symptoms. Here, we describe a case of an elderly male who presented to our clinic with incidental pulmonary nodules and subsequent workup revealed low grade B cell non-Hodgkin's lymphoma consistent with diagnosis of primary pulmonary marginal zone lymphoma.

## 1. Introduction

Primary pulmonary lymphoma (PPL) is a rare neoplasm accounting for 0.5-1% of pulmonary malignancies [1]. It can present with nonspecific symptoms or, in some cases, it can be detected as an incidentaloma during surveillance of other thoracic pathologies. Here, we present a case of an elderly male who was seen at his primary care physician's (PCP) office for follow-up of ascending aortic aneurysm. Computed tomography (CT) of the chest without contrast revealed pleural-based right upper lobe, central right lower lobe, and left upper lobe pulmonary nodules. Pathology confirmed the diagnosis of low-grade B-cell non-Hodgkin's lymphoma (NHL) consistent with marginal zone lymphoma.

## 2. Case Presentation

**Figure 1:**
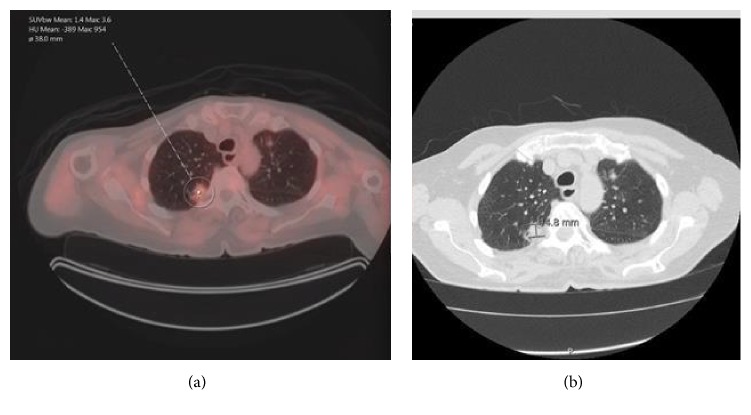
(a) On the left of the picture and (b) on the right of the picture. (a) PET CT showing increased uptake in the nodule. (b) CT Chest without contrast: 14.8 mm pleural-based mass-like density in the posterior medial right upper lobe.

**Figure 2:**
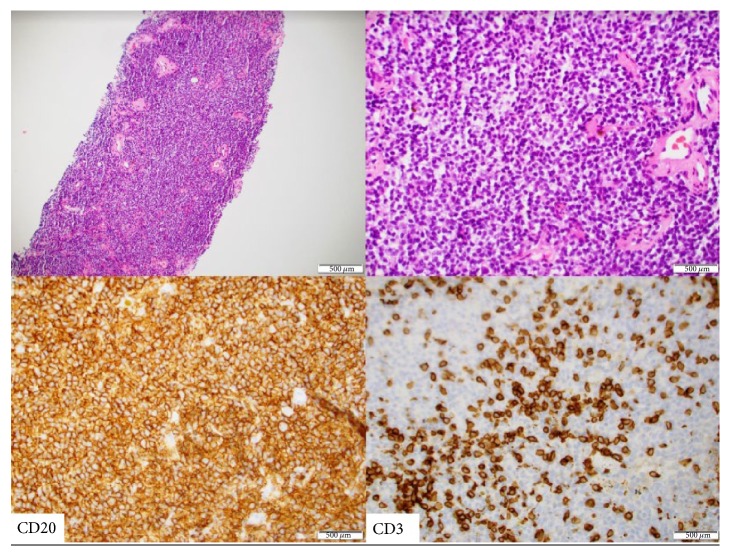
The two pathology pictures are low and high power of hematoxylin and eosin-stained slides showing proliferation of small lymphocytes, a few of them exhibiting monocytoid morphology. The bottom left is CD20 immunostaining highlighting the neoplastic B cells. Bottom right is CD3 immunostaining highlighting the background benign small T cells consistent with diagnosis of marginal zone lymphoma.

An 83-year-old male with past medical history of mild Alzheimer's dementia, nonischemic cardiomyopathy, aortic regurgitation, and an ascending aortic aneurysm measured at 4.1 cm presented to the pulmonology clinic with an abnormal CT of the chest. He was seen by his PCP recently for follow-up of ascending aortic aneurysm and had a CT chest without contrast performed which showed 14.8 mm pleural-based nodular density in the posterior medial right upper lobe, irregular 20.5 mm right lower lobe nodule, and a 2 mm left upper lobe partially calcified nodule [Figure 1(a)]. He had no respiratory or constitutional symptoms. He was a lifelong nonsmoker without any significant occupational exposures. [^18^F]Fluorodeoxyglucose-positron emission tomography with CT (^18^F –FDG PET/CT) was performed showing multiple ^18^F –FDG avid nodules in the right upper lobe, right lower lobe, and left upper lobe [Figure 1(b)]. Initially thought to be inflammatory in nature, a 3-month follow-up CT chest was recommended; however, the patient opted for CT-guided transthoracic biopsy. Because the tissue sample was a core needle biopsy of a pleural-based nodule, it did not include any bronchial epithelium; therefore, the presence of lymphoepithelial lesions could not be evaluated. Immunohistochemical staining showed CD20 positive neoplastic B cells with CD3 positive small benign T cells. CD21 stain was also positive within the residual dendritic cell network, such that the marginal zones appeared to be expanded. These findings support a histopathologic diagnosis of low-grade B-cell NHL most consistent with marginal zone lymphoma [Figure 2]. The patient was referred to oncology for further management.

## 3. Discussion

Primary pulmonary lymphoma (PPL) is a rare clinicopathologic entity, which comprises less than 0.3% of all primary lung malignancies, less than 1% of all cases of NHL, and 3% to 4% of all extranodal NHL [2–4]. It is defined as a lymphoma localized to the lung in a patient with no prior history of extrapulmonary disease at the time of diagnosis or up to 3 months thereafter [2, 5].

The most common type of PPL is the marginal zone lymphoma (MZL) of mucosa-associated lymphoid tissue (MALT), otherwise known as MALT lymphoma or “MALToma,” which comprises 70-80% of all cases of PPL [6]. These MALT lymphomas are thought to arise from clonal proliferation of marginal zone B cells of bronchial-associated lymphoid tissue (BALT) [2]. Approximately 40-50% of pulmonary MALT lymphomas are positive for t(11;18)(q21;q21) [7]. This unique translocation is responsible for the creation of a fusion RNA transcript from the* API2 (apoptosis inhibitor 2) *and the* MALT1* genes which induces activation of the NF-*κ*B pathway resulting in cell proliferation [8, 9]. Unlike gastric MALT lymphoma which is associated with* Helicobacter pylori*, MALT lymphoma of the lung has not been linked to any infectious or specific autoimmune conditions, although there have been case reports associated with tuberculosis [10]. Like gastric MALT lymphoma, it is postulated that pulmonary MALT lymphoma can progress to high-grade diffuse large B-cell lymphoma (DLBCL); however, this has not been well studied [1].

The clinical presentation is highly variable. Most patients are clinically asymptomatic or present with constitutional symptoms, cough, hemoptysis, or dyspnea; B-symptoms are uncommon. Median age at time of diagnosis is 60 years, however, it has also been diagnosed in younger individuals who are usually immunocompromised [2]. One-third of patients have concurrent autoimmune conditions such as rheumatoid arthritis, Sjögren's syndrome, and systemic lupus erythematosus and up to 40% of patients have monoclonal gammopathy as well [11].

Radiographic appearance is variable, ranging from consolidations to inconspicuous nodules or masses. The presence of ground glass opacities, air bronchograms, and bronchiectasis may confound the diagnosis suggesting an infectious or inflammatory process. Presence of air bronchograms is due to relative airway-sparing nature of the disease [2]. Hilar lymphadenopathy is present in approximately 30% of cases [12].

Histologic confirmation is required for definitive diagnosis and is characterized by reactive lymphoid follicles with diffuse infiltration by small lymphocytes and lymphoid proliferation which leads to expansion of the marginal zone; lymphoepithelial lesions (infiltrates of 5 or more neoplastic B-cells into the bronchial epithelium) support the diagnosis but are not required for diagnosis [2]. Lymphoepithelial lesions can be detected with immunohistochemical stains for cytokeratin (which highlights epithelial cells only but not lymphocytes) and CD20 (which highlights lymphocytes but not epithelial cells). As the MALT lymphoma grows, the bronchial wall and adjacent lung parenchyma can be replaced but necrosis or airway obstruction is rare [2].

Primary pulmonary MALT lymphoma is associated with a good prognosis; 5-year and 10-year survival rates are 90% and 70%, respectively [13, 14]. Currently, there are no established guidelines for the management of pulmonary MZL. Considering the indolent course of disease, observation and treatment for symptomatic disease are both reasonable options. Treatment can be considered for symptomatic patients. Therapeutic options include surgical resection, chemotherapy, and radiation therapy. Localized or peripheral lesions can be treated with surgical resection or moderate-dose radiation therapy [15, 16]. Patients with widespread disease not amenable to resection may be treated with single agent chemotherapy, such as rituximab, which has been shown to be effective [17]. Combination chemotherapy with cyclophosphamide, vincristine, and prednisone (CVP) can be considered as well [18]. There is no consensus on whether surgical resection is associated with better outcomes. Further prospective clinical research is greatly needed to determine the optimal treatment modality.

## 4. Conclusion

Nonspecific presentation and indolent course make the diagnosis of primary pulmonary lymphoma very challenging and often lead to misdiagnosis or delayed diagnosis. This diagnosis should especially be suspected in individuals who present with lung nodules but lack usual risk factors for primary non-small-cell and small-cell lung cancer, similar to our patient.
